# Pain Sensitivity, Negative Affect, and Alcohol Use Disorder Status: A Moderated Mediation Study of Emotion Dysregulation

**DOI:** 10.3390/jcm10061321

**Published:** 2021-03-23

**Authors:** Maciej Kopera, Elisa M. Trucco, Hubert Suszek, Paweł Kobyliński, Paweł Wiśniewski, Marcin Wojnar, Andrzej Jakubczyk

**Affiliations:** 1 Department of Psychiatry, Medical University of Warsaw, 00-665 Warsaw, Poland; maciej.kopera@wum.edu.pl (M.K.); marcin.wojnar@wum.edu.pl (M.W.); ajakubczyk@wum.edu.pl (A.J.); 2 Department of Psychology, Center for Children and Families, Florida International University, Miami, FL 33199, USA; etrucco@fiu.edu; 3 Department of Psychiatry, Addiction Center, University of Michigan, Ann Arbor, MI 48109, USA; 4 Faculty of Psychology, University of Warsaw, 00-183 Warsaw, Poland; hubert.suszek@psych.uw.edu.pl; 5 National Information Processing Institute, Laboratory of Interactive Technologies, 00-608 Warsaw, Poland; pawel.kobylinski@opi.org.pl

**Keywords:** alcohol use disorder, pain sensitivity, negative affect, emotional regulation

## Abstract

Previous work suggests that the association between pain and emotional processes among individuals with alcohol use disorder (AUD) may differ from healthy controls. This study investigates whether pain sensitivity mediates the association between negative affect and emotional dysregulation and whether this association differs across AUD status using moderated mediation. The sample included 165 individuals diagnosed with AUD and 110 healthy controls. Of interest was pain sensitivity, as assessed with the Pain Sensitivity Questionnaire, negative affect, as assessed with the Beck Depression Inventory, and emotional dysregulation, as assessed with the Difficulties in Emotional regulation Scale. Age, biological sex, and current pain severity were included as covariates. The results support a moderated partial mediation model that explained 44% of the variance in emotional dysregulation. The findings indicate that negative affect is related to higher pain sensitivity across groups. Moreover, pain sensitivity partially mediated the association between negative affect and emotional dysregulation, but in opposite directions depending on AUD status. Among healthy controls, greater pain sensitivity was related to better emotional regulation, while greater pain sensitivity led to greater emotional dysregulation among individuals with AUD. The potential parallels in the underlying neurobiological mechanisms of emotionality, pain, and AUD suggest that interventions targeting pain may improve adaptive affect regulation skills, which in turn could reduce negative affect and its effect on pain sensitivity among individuals with AUD.

## 1. Introduction

Prior work has consistently established associations between heavy alcohol use and physical pain [1,2]. Namely, physical pain contributes to the development and more severe course of alcohol use disorder (AUD; [1,3]), whereas a reduction of physical pain reduces the risk of relapse in individuals with AUD [4]. Above the simple analgesic effect of alcohol use, the notable co-occurrence of AUD and chronic pain results from the overlapping neurocircuitry underlying pain and substance use disorders. The strong biological evidence of overlapping neurocircuitry led Egli and colleagues [5] to argue that AUD could be conceptualized as either a chronic pain disorder or a type of chronic emotional pain syndrome.

The gate control theory of pain introduced by Melzac and Wall [6] initiated the era of intensive pain research, namely, investigating mechanisms linked to increased and decreased sensitivity of the nociceptive system, depending on the salience of the stimulus. The theory offered a framework for understanding pain as a multidimensional psychophysiological phenomenon with numerous sensory, affective, and cognitive components. Among the psychological mechanisms that influence pain, emotional processes have been found to play a significant role. For example, numerous studies support associations between pain and stress, pain and negative affect, and emotional regulation and pain [7,8,9]. Individuals high in negative affect were found to report (1) higher experimentally induced pain sensitivity (e.g., [10,11]), (2) attenuated pain thresholds (e.g., [12]), and (3) greater activation in brain regions that process emotions (the amygdala and ventrolateral prefrontal cortex) and evaluate sensory processes (anterior insula) [13,14]. Sensory and affective components jointly contribute to the multifaceted experience of pain, yet, it is acknowledged that pain can be experienced in the absence of nociception (i.e., with no sensory stimulation of nociceptive afferents), and can be derived solely from emotional or social sources [15].

In the context of the pain–emotion link, an important distinction should be made between the acute versus the chronic nature of pain. In healthy controls experiencing acute pain, negative mood increases pain sensitivity [8,16] via the process of sensitization of an individual’s attentional resources towards sensory/painful events [17]. In order to keep homeostasis, the brain’s response to rewarding versus painful stimuli within the mesolimbic dopamine system drives the organism to seek reward-oriented behaviors and avoid those that cause acute pain or negative affect (anxiety or depression). Under this framework, if activation within brain regions responsible for emotional regulation and other self-regulation strategies is sufficient to offset activation in systems involved in stress, the organism may regain homeostasis [18,19]. This bidirectional pain–emotional regulation association is in line with research showing that maladaptive emotional regulation strategies are associated with heightened physiological responses [20]. Accordingly, Ruiz-Aranda and colleagues [21] showed that healthy women with better emotional regulation skills perceived a standard pain stimulus as less painful than did women who self-reported deficits in emotional regulation.

In chronic pain, the central sensitization phenomenon manifests as pain hypersensitivity, triggered by a prolonged increase in the excitability and synaptic efficacy of neurons in central nociceptive pathways [22]. Consequently, physical and social functioning impairment amplifies negative emotional states [23], leading to emotional dysregulation, which fuels negative affect instead of releasing it, as in the case of acute stress. Difficulties with emotional regulation have been consistently shown to be associated with chronic pain conditions (e.g., [24,25,26]). Moreover, studies indicate that individual differences in emotional regulation impact the association between pain and negative affect [27].

Negative affect has been consistently found to be a significant contributor to AUD development [28] and a leading precipitant of relapse [29]. According to Koob and Le Moal during acute withdrawal and protracted abstinence, persistent dysregulation of the activity of neural circuits that mediate motivated behavior leads to the development of an allostatic state: “a state of chronic deviation of the regulatory system from its normal (homeostatic) operating level” [30]. At this point, decreases in reward function and increases in stress function (negative reinforcement) contribute to both negative emotionality and hypersensitivity for pain [19]. As the allostatic load grows larger, negative emotions and pain hypersensitivity (as in chronic pain) fuel negative reinforcement and motivate alcohol drinking in order to manage pain and negative emotions simultaneously.

According to Gross [31], emotional regulation is an adaptive ability to modulate the experience of emotion in order to achieve a desired goal in a certain environmental context. Through emotional regulation, an individual can “influence which emotions one has, when one has them, and how one experiences or expresses these emotions” [32]. The ability to adaptively regulate emotional experience is crucial for both mental and physical well-being. Current research supports a strong contribution of emotional dysregulation in the etiology of substance use [33,34]. Although the acute effect of alcohol on emotional processes might be reinforcing, the chronic effect can be detrimental to emotional regulation neurocircuitries, and, in the long run, fail to alleviate negative affect while fueling the allostatic state [28,35]. Importantly, emotional regulation is considered to be involved in the relation between negative affect and pain in individuals with AUD. In a study by Kopera at al. [36], emotional regulation appeared to fully mediate the association between depression severity and pain severity in an AUD sample. Recently, Jakubczyk and colleagues [37] reported that the association between interoceptive accuracy (i.e., precision in perceiving internal processes measured behaviorally) and pain sensitivity may be moderated by AUD status. Namely, while interoceptive accuracy was associated with lower pain sensitivity among individuals with AUD, the opposite was true among healthy individuals. Given that better interoceptive accuracy is positively associated with emotional regulation [37], it is plausible that the association between pain sensitivity and emotional regulation could differ across AUD and non-AUD samples.

Taken together, previous work suggests that the association between pain and emotional processes among individuals with AUD may differ compared to healthy controls, and yet, these associations have not been investigated to date. The aim of the current study was to assess whether pain sensitivity mediates the effect of negative affect on emotional dysregulation and whether differences exist across AUD status using moderated mediation modeling. It was hypothesized that greater negative affect (i.e., depressive symptom severity) would be associated with increased self-reported pain sensitivity across the entire sample. Yet, it was also hypothesized that the association between pain sensitivity and emotional dysregulation would differ across AUD status. The consequence of higher pain sensitivity induced by negative affect in non-AUD individuals without a history of chronic pain was expected to increase sensitivity to potentially harmful stimuli, and increase emotional regulation in order to use emotional coping to react to the environmental threat. It was expected that this same adaptation mechanism would not operate among individuals with AUD. Namely, once pain sensitivity is increased by negative affect, the negative reinforcement mechanisms associated with alcohol use would be expected to maintain the allostatic state and outweigh alternative self-regulation strategies, leading to greater emotional dysregulation.

## 2. Materials and Methods

### 2.1. Participants

The data come from an ongoing study examining emotional and behavioral functioning among individuals treated in an inpatient setting for AUD and a comparison sample of healthy controls (HCs). The study sample consisted of 165 adults (44 ± 11.2 years of age) admitted to an eight-week, drug-free, abstinence-based, inpatient alcohol treatment program. HCs included 110 adults (40.6 ± 8.1 years of age) that met with a primary care physician for medical advice or a yearly physical examination. Jakubczyk and colleagues [37] provide an additional description of the sample with respect to sociodemographic information and clinical and alcohol use characteristics. The AUD sample consisted of individuals with severe symptoms of AUD, but the absence of acute withdrawal symptomatology, with an average duration of 49.2 ± 45.1 days abstinent from alcohol prior to completing the study procedures. Study procedures were performed during the first two weeks after treatment admission. Given the overrepresentation of men in substance use treatment programs in Poland, a large portion of the sample was White men (88.1%) in the AUD sample and 74.5% in the HC sample.

AUD diagnosis was obtained through the International Classification of Diseases and Related Health Problems 10th Revision [38] upon treatment admission, and was later confirmed through the MINI International Neuropsychiatric Interview [39]. Exclusion criteria included the following: a clinically significant cognitive deficit (<25 on the Mini-Mental State Examination) [40], a history of psychosis, co-occurring current psychiatric disorders requiring medication, current use of analgesics, and co-occurrence of substance use/dependence other than nicotine. HCs were excluded if they endorsed harmful alcohol use as measured by the Alcohol Use Disorders Identification Test [41]. Participants were 40.6 ± 8.1 years of age. When comparing groups across demographic characteristics, HCs were significantly younger (*F*(266) = 7.65, *p* = 0.006) and less likely to be male (χ^2^ (1, 269) = 8.2, *p* = 0.004) compared to the AUD sample. Thus, age and biological sex were included as covariates in all analyses.

The study was conducted in accordance with the ethical principles described in the Declaration of Helsinki in 1964, and received approval from the Bioethics Committee of the institution where the study took place.

### 2.2. Measures

- Sociodemographic. Questions regarding sociodemographic characteristics (e.g., age, biological sex, education) were obtained using a self-report questionnaire.
- Alcohol use factors. The Short Inventory of Problems [42] was used to quantify the number of consecutive days of heavy drinking, maximal amount of alcohol consumed during periods of consecutive heavy drinking, and period of abstinence prior to the assessment via an interview. The age of drinking problem onset from the modified version of the Substance Abuse Outcomes Module [43] was used to determine the duration of problematic alcohol use as self-reported by individuals with AUD.
- Pain sensitivity. Pain sensitivity was assessed with the Polish version of the Pain Sensitivity Questionnaire (PSQ; [44,45]). The PSQ is a self-reported measure of pain sensitivity that was shown to be strongly correlated with experimental pain intensity rating measures [44,46], and a clinically sensitive predictor of postoperative pain severity [47]. This is a 17-item measure reflecting self-reported pain sensitivity in everyday situations (e.g., “Imagine you bump your elbow on the edge of a table.”). Three items describe non-painful situations. Thus, the PSQ score reflects an average of 14 items [44]. Participants are asked to rate how painful each situation would be on a scale of 1 (not at all painful) to 10 (most severe pain imaginable). The Cronbach’s α for this scale was 0.92.
- Negative affect. The total score of the Beck Depression Inventory II [48] utilized as a measure of current negative affect severity.
- Emotional dysregulation. The Polish version of the Difficulties in Emotional regulation Scale (DERS) [49,50] was used to assess emotional dysregulation. The DERS reflects emotional dysregulation across six domains: non-acceptance of negative emotions, inability to engage in goal-directed behaviors when experiencing negative emotions, difficulties controlling impulsive behaviors when experiencing negative emotions, limited access to effective emotional regulation strategies, and lack of own emotional awareness and clarity. A total DERS score was used for the current study (Cronbach’s alpha = 0.93). Higher scores on the DERS indicate worse emotional regulation.
- Current experience of pain. A visual analogue scale (VAS) was used to assess pain. Participants were asked to make a mark on a 10 cm horizontal line ranging from “no pain at all” to the “worst pain imaginable”, reflecting their current experience of physical pain. This measure provided confirmation that individuals with AUD report more overall severe current pain in comparison to HCs (see [37]). Moreover, the current experience of pain was included as a covariate.

### 2.3. Data Analysis

**Figure 1:**
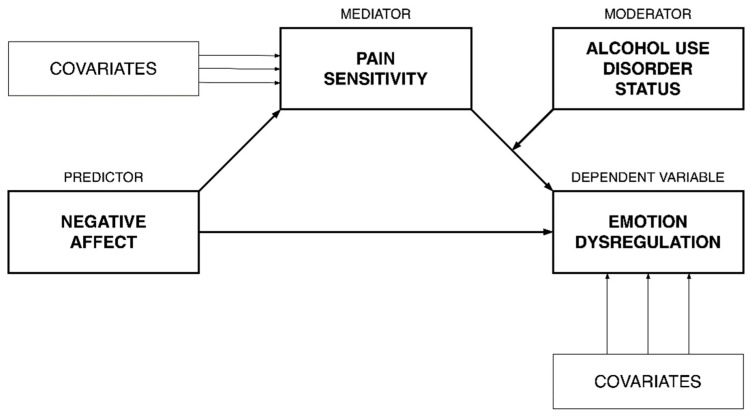
Conceptual diagram for the moderated mediation model.

In order to test AUD status as a potential moderator of the mediating effect of pain sensitivity on the association between negative affect (depression severity) and emotional dysregulation, Hayes’ (2018) PROCESS SPSS macro for moderated mediation with bootstrapping (5000 resamples with replacement) was applied (see Figure 1 for a conceptual model). More specifically, pain sensitivity was included as a mediator in the association between depressive symptom severity on emotional dysregulation, with AUD status included as a moderator of the second link (i.e., the association between pain sensitivity and emotional dysregulation). Age, biological sex, and current pain severity were included as covariates. Simple slope analyses reflecting a “pick-a-point” approach were conducted to probe significant interactions. This consists of a set of regression tests that determine where in the distribution of the moderator the predictor (the mediator in this case) has an effect on the dependent variable [51]. Non-standardized coefficients are reported throughout the paper.

## 3. Results

**Figure 2:**
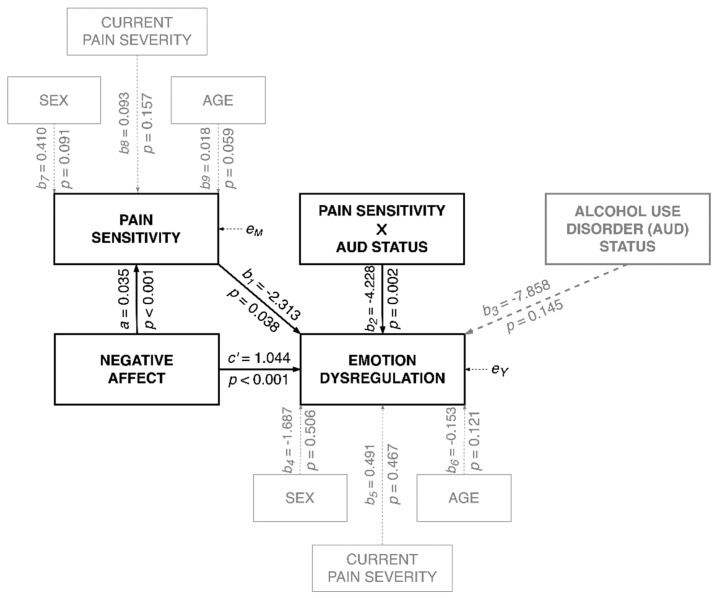
The moderated mediation model for emotional dysregulation.

**Figure 3:**
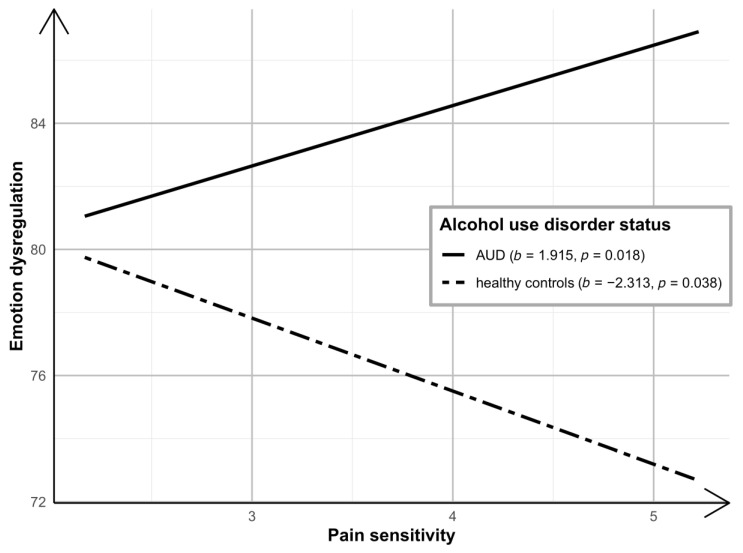
Pain sensitivity effect on emotional dysregulation by AUD status.

The moderated mediation model with negative affect as the predictor, pain sensitivity as the mediator, AUD status as the moderator, and emotional dysregulation as the dependent variable was tested (see Figure 2 for non-standardized coefficients). The results supported moderated partial mediation. The model explained 9% of the variance in pain sensitivity (*R*^2^ = 0.086; *F*(4247) = 5.824; *p* < 0.001) and 44% of the variance in emotional dysregulation (*R*^2^ = 0.439; *F*(7244) = 27.262; *p* < 0.001). There was support for a significant two-way interaction between pain sensitivity and AUD status on emotional dysregulation (*b* = 4.228; 95% CI = (1.538, 6.919); *p* = 0.002. Δ*R*^2^ = 0.022). As depicted in Figure 3, findings indicate that the simple slopes for the regression of emotional dysregulation on pain sensitivity were statistically significant for both HCs (*b* = −2.313; 95% CI = (−4.502, −0.125) *p* = 0.038) and individuals with AUD (*b* = 1.915; 95% CI = (0.329, 3.501]; *p* = 0.018). Yet, emotional dysregulation was negatively associated with pain sensitivity among HCs, and positively associated with pain sensitivity among individuals with AUD. The conditional indirect effect of negative affect on emotional dysregulation via pain sensitivity was significant for both HCs (*c_HC_* = −0.081; bootstrap 95% CI = (−0.183, −0.011)) and individuals with AUD (*c_AUD_* = 0.067; bootstrap 95% CI = (0.0001, 0.154)). In general, greater negative affect was associated with more pain sensitivity. However, while emotional dysregulation was indirectly (via pain sensitivity) negatively associated with negative affect among HCs, it was also indirectly positively associated among individuals with AUD. That is, greater pain sensitivity was associated with less emotional dysregulation among HCs, but more emotional dysregulation among individuals with AUD. Moreover, the indirect effects were significantly different (index of moderated mediation: *c_AUD_*-*c_HC_* = 0.147; bootstrap 95% CI = (0.042, 0.293)).

## 4. Discussion

The current study is among the first to investigate whether pain sensitivity mediates the association between negative affect and emotional dysregulation, and if this association differs across AUD status using moderated mediation. Consistent with the hypotheses, the findings indicate that, in both groups, negative affect (current depressive symptom severity) is related to higher pain sensitivity. Moreover, AUD status was found to significantly moderate the association between pain sensitivity and emotional dysregulation. That is, pain sensitivity partially mediated the association between negative affect and emotional dysregulation, but in opposing directions based on AUD status. Among healthy individuals, pain sensitivity was negatively associated with emotional dysregulation (i.e., higher pain sensitivity leads to better emotional regulation; see Figure 3). In contrast, among individuals with AUD, the association between pain sensitivity and emotional dysregulation was in the opposite direction (i.e., higher pain sensitivity leads to higher emotional dysregulation; see Figure 3). These results extend prior findings demonstrating between-group differences across individuals with AUD and HCs in the association between interoceptive and pain sensitivity [37].

### 4.1. Negative Affect, Pain Sensitivity, and Emotional Dysregulation in Healthy Individuals

Consistent with prior work among HCs (or individuals that do not experience chronic pain), the findings supported an association between depressed mood and increased pain sensitivity [8,16]. Neurobiologically, signaling of acute pain serves as a protective factor that enables fast and effective removal or avoidance of harmful stimuli. In order to keep homeostasis, the brain’s response to painful stimuli within the mesolimbic dopamine system motivates the organism to avoid behaviors that cause pain or negative affect. Previous findings in HCs showed that there might be a sensitization mechanism activated by negative mood that distributes attentional resources towards upcoming sensory/painful events [17]. This adaptation mechanism, the purpose of which is to detect potentially threatening environmental events, is fundamental for survival, and allows for faster and more adequate self-regulation [18]. Findings have consistently demonstrated that, in healthy individuals, increased pain sensitivity is related to better emotional regulation. Moreover, in the current study, there is evidence for a positive and significant association between negative affect and emotional dysregulation. Accordingly, lower emotional dysregulation among HCs may attenuate negative affect and refine the homeostatic mechanism in search of balance.

Prior work finds that the PSQ is significantly correlated with experimental pain intensity ratings [44], suggesting that pain sensitivity may be interpreted as a reliable measure of general, non-acute pain intensity. In the current study, the need to decrease pain sensitivity in HCs may not be a priority, as they experience significantly lower pain sensitivity in comparison to individuals with AUD. In non-AUD individuals, alcohol remains a well-known analgesic, and it does decrease pain sensitivity, but at the same time, it disrupts emotional regulation [52]. Therefore, in acute pain, even if alcohol drinking can alleviate the experience of its physical component, it does not enhance emotional regulation and cannot be considered an efficient regulative strategy to cope with more complex (physical and emotional) pain experiences [53].

### 4.2. Negative Affect, Pain Sensitivity, and Emotional Dysregulation in Individuals with AUD

Although greater pain sensitivity might be adaptive in the case of acute stress, such as pain, when a stressor can be immediately removed, it becomes maladaptive as higher pain sensitivity transitions into a chronic experience, as observed among individuals with AUD. Moreover, bidirectional associations between increased negative emotions and pain sensitivity may have the same neurobiological underpinnings, as some other authors note in previous research [54,55]. The conceptualization proposed by Lane et al. [56], based on work with psychosomatic patients, suggests that a focus on physical pain may serve as a type of replacement for emotional pain that is less tolerable. Similar to psychosomatic patients, individuals with AUD in the current study are characterized by high alexithymia (see previous report by [57]). That is, these individuals may easily direct their attention on the physical manifestation of emotional arousal instead of the feeling of negative emotion. Although the experience of pain may serve as a temporary beneficial “psychic regulator” once it provides avoidance of unbearable emotions (see [56] for review), individuals with AUD may choose an alternative solution to emotional regulation, which is alcohol use. Such conditions may drive alcohol consumption in an effort to regulate one’s emotional state and restore the organism to a more natural hedonic/emotional state [58].

According to the opponent processes theory, individuals with AUD gradually become tolerant to the initially rewarding effects of alcohol. At the same time, they may also become increasingly sensitized to negatively reinforcing aspects of alcohol use, including the relief of withdrawal symptoms [28]. Notably, Jochum and colleagues [59] confirmed in humans a withdrawal-induced increase in sensitivity towards painful stimuli. Repeatedly occurring episodes of alcohol intoxication and withdrawal may alter brain stress circuitry (including corticotropin-releasing factor signaling), and are thought to stimulate a persistent negative mood, including hypersensitivity to pain [60]. Individuals learn to detect internal cues of negative affect (such as pain) while experiencing periods of withdrawal. They in turn may respond to this by taking the drug again [61]. Maintenance of allostatic load in protracted abstinence is supposed to keep individuals with AUD within an allostatic state [28]. Once the learning process is strengthened by periods of repeated withdrawal, individuals with AUD become more sensitive to somatic/emotional negative cues, and drug use becomes an avoidance strategy that allows the immediate change in affective and physiological states without the need to address the specific issue. If lower pain sensitivity is associated with lower emotional dysregulation (as demonstrated among individuals with AUD), then, among individuals with AUD, a decrease in pain sensitivity may consequently downregulate negative affect. Importantly, the current AUD sample is characterized by significantly higher pain sensitivity in comparison to HCs. Therefore, it is likely that these individuals may use alcohol in order to decrease pain sensitivity and enhance emotional regulation skills, achieving a temporary decrease in negative affect and both somatic and emotional well-being. As such, within the AUD sample, alcohol may be viewed as an efficient regulative strategy to cope with complex (physical and emotional) experiences of pain. Upon repeated strengthening of this maladaptive coping strategy over more adaptive affect regulation solutions, emotional regulation difficulties may increase, thus enhancing the appeal of alcohol drinking that characterizes the vicious cycle of the disorder. This hypothesis has been supported by the results of Lutz and colleagues [26], where they found that greater emotional dysregulation was related to greater risk for opioid misuse in individuals with chronic pain. Thus, among individuals with AUD, higher pain sensitivity no longer characterizes better adaptation. Drinking becomes driven more by the motivation to avoid or regulate negatively balanced and/or physically painful consequences of withdrawal, thus feeding “the dark side” of addiction [28]. At this point, negative affect tends to prevail, leading to increased pain sensitivity and pain-related emotional dysregulation that concurrently fuel the experience of negative affect.

A greater understanding of the association between pain and emotional regulation may help develop additional targets for clinical interventions. Plausibly, AUD treatment programs could benefit from working on strategies that specifically address associations between pain, negative affect, and emotional dysregulation. The efficacy of psychological interventions targeting pain and improving functioning in persons with a broad spectrum of pain-related conditions has been established [62,63]. However, these treatments have not been thoroughly examined or adopted among individuals with AUD. Mindfulness has been suggested as a potentially beneficial therapy for chronic pain [62], and has also been used for relapse prevention in patients with AUD [64]. Decreases in pain ratings among individuals receiving cognitive behavioral therapy (CBT) for pain management [65] and increases in self-efficacy to manage pain were seen in patients treated for substance use disorders [66]. This intervention involves developing skills to cope with pain, promoting abilities to foster acceptance of pain, and enhancing cognitive techniques that may reduce the experience of pain [65,67,68]. Future research could prospectively assess the efficacy of interventions combining adaptive affect regulation skills and pain sensitivity on the course and treatment outcomes among individuals with AUD.

The current study has important limitations that should be noted. This study is cross-sectional, so the direction of effects cannot be established, and tested pathways are not causative. Recruitment only consisted of individuals entering an inpatient treatment program for a severe course of AUD. Therefore, emotional regulation and pain sensitivity may have been impacted by heavy alcohol use, and results may not generalize to less severe AUD cases. Participants in the AUD group were significantly more likely to be male and older in comparison to healthy controls. Although age and sex were used as covariates in all analyses, the older age of individuals with AUD may have influenced the results, as age is associated with higher pain sensitivity among individuals with AUD due to emerging polyneuropathy. Unfortunately, detailed data regarding the duration of pain were not available within our sample. Yet, given the fact that the AUD group was characterized by a long history and high severity of drinking problems (which likely led to somatic diseases), the pain experienced by this group may have been chronic rather than acute in nature. Moreover, the reliability of retrospective self-reported data from heavy drinking individuals with AUD might have been compromised by the analgesic effect of alcohol, as 70% of individuals in the AUD sample considered alcohol to be an effective painkiller. Moreover, the study focused on self-report assessments of pain sensitivity. Future work should also utilize behavioral measures of pain threshold and account for the duration of experienced pain.

## 5. Conclusions

The results indicate that pain sensitivity mediates the effect of negative affect on emotional dysregulation, and that the link between pain sensitivity and emotional dysregulation differs across AUD status. The potential parallels in the disruption of balance during the progression from acute to chronic pain or from otherwise non-problematic alcohol use to AUD strengthens the possibility of common underlying neurobiological mechanisms [18]. Future work should seek to gain a deeper understanding of this association and investigate its possible therapeutic significance and implications.

## Data Availability

Data are available on request from the corresponding author.
